# Supplementary material for: Are carbon emissions trading and green financial instruments synergistic? -Comprehensive quantitative research based on content analysis
Source: PLoS One. 2024 Mar 7;19(3):e0298601. doi: 10.1371/journal.pone.0298601 (PMC10919601; doi:10.1371/journal.pone.0298601)
Supplement: S1 Data — (ZIP) [file pone.0298601.s001.zip › Data/地级市Z_F工作报告.zip!/╔╜╢1⁄2/╡┬╓▌╩╨/└┤╫╘╣1⁄2╓┌║┼í╛┬φ┐╦╩2╛▌═°í┐_2016.docx]

各位代表：

现在，我代表市人民政府向大会报告工作，请予审议，并请市政协委员和其他列席会议的同志提出意见。

一、“十二五”经济社会发展情况

2011年至2015年，在中共德州市委的正确领导下，在市人大、市政协及社会各界的监督支持下，我们紧紧依靠全市人民，万众一心，扎实工作，奋力推进经济建设、政治建设、文化建设、社会建设、生态文明建设，实现了“十二五”胜利收官。

五年来，城市综合实力迈上新台阶。2015年，全市地区生产总值实现2751亿元，是2010年的1.7倍;公共财政预算收入182.8亿元，是2010年的2.5倍;固定资产投资、社会消费品零售总额、外贸出口分别达到2238亿元、1257亿元和22.1亿美元，分别是2010年的2倍、1.9倍和1.1倍;金融机构存、贷款余额2470.3亿元和1546亿元，分别是2010年的1.9倍和1.7倍。三次产业占比由2010年的12.7:54.3:33调整为10.3:49.4:40.3，呈现出结构不断优化、质量不断提升、协调性逐步增强的良好态势。

五年来，人民生活水平大幅提高。全市城镇居民人均可支配收入达到21039元(新统计口径)，年均增长11.1%，农村居民人均可支配收入达到 11269元(新统计口径)，年均增长13.6%，城乡收入比由2.09:1缩小到1.87:1。农村贫困人口减少41万人。人均预期寿命达到76岁左右。新建改建学校304所，基础教育全面加强，职业教育蓬勃发展。城镇人均住房面积达到39.48平方米，增加7.61平方米;农村人均住房面积达到43.5平方米，增加9.48平方米。城镇每百户拥有汽车由20.5辆提高到46.39辆，农村由5.1辆提高到19.09辆。农村自来水普及率达到97%，基本实现城乡同网同质供水。信息消费大幅增加，家庭固定宽带普及率提高30个百分点，达到45%。

五年来，实体经济加快发展。工业转型升级步伐加快，质量效益显著提高，全市规模以上工业企业达到2930家，规模以上工业增加值年均增长14.2%，高新技术产业产值占比提高7.4个百分点。民营经济蓬勃发展，竞争力不断增强。县域经济综合实力明显提升，7个县(市、区)公共财政预算收入过10亿元，齐河县跻身全国百强县。深入实施创新驱动战略，科技金融产业融合发展成效显著，积极开展大院大所对接活动，转化科技成果305项，中科院综合技术转化中心、教育部“蓝火计划”等落户我市。成功举办五届区域性资本交易大会，163家企业完成股份制改造，上市挂牌企业达到117家。德州经济技术开发区和高新技术产业开发区晋升为国家级，获批中国新能源和生物产业引智试验区。农业现代化水平稳步提升，粮食生产实现“十三连增”，农业合作化水平不断提高，农民合作社达到12969家，土地流转面积占耕地总量的31.1%，获批国家现代农业示范区。现代服务业加快发展，万达广场、银座商城、泉城欧乐堡、德百温泉度假村等一批商贸文化旅游项目建成运营，电子商务年交易额突破700亿元。

五年来，城乡统筹迈出新步伐。城市功能日臻完善，基础设施建设取得新跨越，实施重点城建工程412项，建成大外环、“两河四岸”、开发区路网等城市主干交通框架，京沪高铁、邯济、德大铁路、德滨、济乐高速建成通车，德上高速、济齐黄河大桥开工建设。陵县撤县设区，中心城区面积扩大，大社区建设取得显著成效。新型城镇化建设加快步伐，“两区同建”模式成为全国典型，常住人口城镇化率达到51.73%，提高8.68个百分点。县城和15个示范镇建设稳步推进，新建改造农村公路6900公里，城乡环卫一体化基本实现全覆盖，城镇污水集中处理率达到94.17%，提高13.7个百分点。南水北调东线德州段工程顺利竣工，11座平原水库建成使用。节能减排目标顺利完成，建成人工湿地2.8万亩，林木覆盖率达到35%。

五年来，重点领域改革全面推开。22项国家级、30项省级改革试点顺利实施。积极推动大众创业、万众创新，深化商事制度改革，新注册市场主体13.5 万户，年均增长19.3%。卫生计生、食品药品、文化、工商、质监等机构改革全面完成。规范透明的预算制度基本建立，市和县(市、区)级预算全部公开。金融改革创新力度加大，新引进商业银行7家，新增保险公司13家，建立小额贷款、担保等地方金融组织78家;建立了风险预警化解协调机制，不良贷款率下降5.73个百分点;市政府设立5亿元股权投资引导母基金、3亿元政府性融资担保机构，加强了金融调控能力。基本完成农村土地承包经营权确权颁证工作。县级公立医院综合改革全面铺开，城乡居民基本养老保险和基本医疗保险实现整合。

五年来，对外开放和区域合作取得重要进展。成功纳入国家京津冀协同发展和省“一区一圈一带”战略规划，纳入国家重大发展战略规划将对德州长远发展带来重大机遇和深远影响。省委、省政府高度重视，专门出台文件支持德州融入京津冀协同发展。围绕“一区四基地”功能定位，全面深化与京津冀地区的对接合作，与国家相关部委和京津推进京津冀协同发展领导小组办公室建立了工作联系，与京津两市7个区签订战略合作协议。 2015年先后在北京举办两次投资推介会，签订产业项目协议172个，总投资851.6亿元，北汽40万辆、奇瑞10万辆新能源汽车、中广核新能源示范区、中航科工机器人等一批高端产业项目顺利签约。生态科技城被国家列为京津冀产业承接特色园区。国开行、农发行、进出口银行、中国银行等金融机构授信1000亿元支持德州融入京津冀。与中科院、中关村等科研机构建立了有实质内容的合作关系，医疗、教育、文化、旅游等领域的全方位对接合作局面初步形成。全面对接省会发展，72项重点工作顺利推进，山东大学生物技术研究院落户禹城，省立医疗集团与我市开展医疗合作，产业对接合作日益活跃。对外开放扎实推进，外贸进出口额持续增长，实际利用外资 10亿美元，新引进世界500强企业4家，签订对外承包工程合同额7亿美元，恒源石化成功收购马来西亚石油项目，综合保税区、齐河陆路口岸申建有序推进，与英国邓迪市、美国帝王市、新西兰黑斯廷斯市、法国布希圣乔治市缔结友城。

五年来，社会事业和民生建设蓬勃发展。每年实施一批民生实事，解决群众关心的热点难点问题。城镇新增就业35.9万人，农村劳动力转移就业49.6万人。基本实现全民养老和全民医保，企业退休人员、居民基础养老金分别实现“11连涨”和“4连增”。建设校舍276万平方米，补充教师8703名。新增县级社会福利中心8处，养老床位达到1.8万张。建设保障性住房826.55万平方米，解决了9.6万户家庭住房困难问题。市博物馆、大剧院、档案馆启用，大运河成功申遗。平安德州建设取得新进展，信访、安全生产、食品药品安全工作得到加强。民族宗教、妇女儿童、国防动员、人民防空、防震减灾、应急管理、口岸打私、体育、史志、科普、仲裁、慈善等工作取得新成绩。

五年来，社会主义民主政治全面加强。始终坚持市委的正确领导，坚持自觉接受人大及其常委会的监督，加强与政协的民主协商，虚心听取各民主党派、工商联、无党派人士和社会各界意见。健全完善政府决策程序，建立政府法律顾问制度和重大行政决策合法性审查机制。深化行政审批制度改革，公布实施行政审批事项目录清单、行政权力清单、政府责任清单，电子政务和政府信息公开扎实推进。认真开展党的群众路线教育实践活动和“三严三实”专题教育，严守党的政治纪律和政治规矩，坚决落实中央“八项规定”精神，扎实整治“四风”问题。坚定不移推进党风廉政建设和反腐败斗争，全市纪检监察机关共立查案件5816件，党纪政纪处分5601人。

各位代表，五年来，通过全市上下的共同努力，我们办成了一批事关德州长远发展的大事，城市竞争力和影响力不断增强。成功创建国家卫生城市、国家园林城市、全国双拥模范城、全国粮食生产先进市，获批实施国家新型城镇化等22项国家试点。特别是2014年李克强总理视察德州，对我市新型城镇化等工作充分肯定，给予我们极大的鼓舞。这些成绩的取得，是省委、省政府和市委正确领导的结果，是市人大、市政协和社会各界大力支持的结果，是全市人民团结奋斗、拼搏实干的结果。在此，我代表市政府，向全市人民，向各位人大代表、政协委员、离退休老同志，向各民主党派、工商联、无党派人士和各群众团体，向驻德部队官兵，向中央、省驻德单位，向关心支持德州发展的海内外朋友，致以崇高的敬意和衷心的感谢!

在肯定成绩的同时，我们也清醒地看到，当前我市经济社会发展中还存在一些突出问题：发展的思维还需突破，不少瓶颈问题有待解决，全社会的创新精神需进一步激发;发展的质量还不够高，实体经济缺大少强，优结构、增效益、促转型的任务艰巨，稳增长压力依然较大;民生保障水平尚有差距，城乡间教育、医疗等民生事业和基础设施建设不均衡的矛盾依然存在;资源约束趋紧，生态环境亟待改善，特别是大气污染防治迫在眉睫;行政效能有待提高，依法行政的能力需进一步加强，部分干部的精神状态和工作作风尚需改变，不作为、乱作为的现象时有发生等等。对此，我们一定高度重视并认真解决。

二、“十三五”时期经济社会发展的主要任务

从今年起到2020年，是全面建成小康社会的决胜阶段，是建设协同发展示范区的关键时期。根据中央十八届五中全会、省委十届十三次全会和市委十四届八次全会精神，市政府主持编制了《德州市国民经济和社会发展第十三个五年规划纲要(草案)》，提交大会审议。今后五年的总体目标是：

——全面实施协同发展战略。认真落实创新、协调、绿色、开放、共享发展新理念，统筹实施国家和省重大区域发展战略，全力推进三次产业协同发展、城乡之间协同发展、经济社会民生协同发展，协同发展示范区建设取得重大进展。

——继续保持经济中高速增长。全面实施创新驱动战略，坚定不移转方式、调结构，坚定不移做大做强实体经济，发展质量和效益显著提高。全市地区生产总值年均增长8%，达到4000亿元左右，提前实现经济总量比2010年翻一番，公共财政预算收入年均增长10%左右，达到290亿元，固定资产投资年均增长 14%左右，三次产业结构调整为8:47:45。

——不断提升人民生活水平。坚持发展为了人民，不断提高市民的幸福感，城乡居民收入差距持续缩小，城镇和农村居民人均可支配收入年均分别增长9%和10%，达到32000元和18000元。城乡基本公共服务体系更加健全，均等化水平稳步提高。

——持续改善生态环境。坚持“绿水青山就是金山银山”，全力组织生态环保治理攻坚战，重要环境指标明显改善，生产方式和生活方式绿色、低碳水平不断提升，林木覆盖率显著提高，京津南部、山东北部重要生态功能区建设取得阶段性成果。

——全面提升全社会文明程度。社会主义核心价值观更加深入人心，组织开展丰富多彩的“我文明，德州文明”创建活动。城乡公共文化服务体系基本建成，有德州特色的文化产业不断发展壮大。

实现“十三五”规划目标，必须深入贯彻习近平总书记系列重要讲话精神，坚持“四个全面”战略布局，积极适应和引领经济发展新常态，坚持以创新发展为第一动力，以协调发展为内在要求，以绿色发展为重要取向，以开放发展为必由之路，以共享发展为出发点和落脚点，全力打好协同发展、深化改革、精准脱贫、环境治理、民生改善五大攻坚战，努力建设协同发展示范区，确保与全省同步全面建成小康社会。

(一)坚持创新发展，推动实体经济大跨越。

把创新摆在发展全局的首要位置，制定鼓励创新的政策体系，推动大众创业、万众创新，使创新在全社会蔚然成风。突出发展科技服务业，重视科技金融对创新的支撑作用，积极培养技术经纪人队伍，完善技术交易市场，加快建设各类科技孵化器、加速器。支持德州高新区建设，加快培育生物技术等战略性新兴产业，努力打造协同发展的创新引擎。在生态科技城启动建设德州创新谷，以一流的环境打造城市创新新高地。支持德州学院等驻德高校发挥自身优势，开展技术研发和成果转化。支持企业建设研发中心、开展技术合作、组建创新联盟。

抓住人才这个关键要素，全面落实人才政策“黄金30条”，千方百计吸引各类科研人员、专业技能人才、高校毕业生、归国留学生、外国专家等汇聚德州，坚持“政策引人才、平台聚人才、产业兴人才、环境留人才”，加快人才公寓建设，加快引入国际学校、国际医院，建立服务高端人才的长效机制，努力建设鲁北人才改革试验区。

大力提升工业，厚植产业发展新优势，重点发展新能源汽车、节能环保等十大特色优势产业，加快发展生物技术、智能装备制造等六大战略性新兴产业，促进工业经济迈向中高端，规模以上工业增加值达到3000亿元。集中力量抓大扶强，支持重点企业加大技改力度，加快股份制改造，健全治理结构;支持龙头企业进行产业重组并购、加快做大做强;支持有条件的企业走出去、拓展境内外市场，广泛开展对外合作;支持企业在资本市场的拓展，提高资本运作和直接融资能力。用五年的时间，真正培育起一批规模大、效益好、创新能力强的大企业集团。大力发展现代服务业，加快发展研发设计、现代金融等生产性服务业，积极培育文化旅游、健康养老、体育健身等生活性服务业。深入实施“互联网+”行动计划，支持电子商务发展，大力培育经济发展新业态、新优势。

加快转变农业发展方式，以打造周边城市“放心农场”为目标，着力建设国家现代农业示范区。巩固传统农业优势，确保粮食持续高产稳产;高度重视农产品质量安全体系建设，保证老百姓“舌尖上的安全”;用现代产业的思维全要素推动现代农业转型，积极发展多种形式的适度规模经营，加大农业科技投入，优化种植结构，大力培植农业产业化龙头企业，拉长产业链条，推动一产接二产连三产，真正实现由传统农业大市向现代农业强市转型升级。

(二)坚持开放发展，提升经济发展外向度。

德州具有得天独厚的区位优势，我们要坚决破除封闭保守和小成即满的思想，以更开阔的视野、更开放的胸怀，不断推动对外合作，构建开放新格局。抢抓京津冀协同发展重大历史机遇，深入实施“一区一圈一带”战略，全面对接京津，深度融入省会，南北双向借力，聚焦产业承接、科技成果转化、优质农产品供应、高素质劳动力输送、生态功能区建设等领域，坚持招商引资与招才引智并举、产业项目与社会项目并重，精准谋划、精准对接、精准招商，五年内力争引进京津和省会地区各类项目1000个以上，到位资金1000亿元以上，积极对接环渤海、长三角、珠三角城市群的产业资源，开创协同发展新局面。

抓住国家实施“一带一路”战略机遇，大力拓展对外经贸合作，重点引进世界500强企业，提高利用外资的规模和效益，“十三五”期间，全市新引进外资数量要比“十二五”翻一番。举全市之力支持德州经济技术开发区发展，加快产业集聚，加快新城开发，真正成为全市经济发展和对外开放的龙头，未来五年，市经济技术开发区的经济总量要比“十二五”翻一番。积极争取复制上海自贸区政策机制，推进综合保税区创建，支持齐河申建陆路口岸和多式联运海关监管中心。深入实施走出去战略，加强与欧美、日韩、东盟、港澳台等区域的经贸合作，加大招商推介力度，广泛开展教育、文化等方面的合作，加强与国际友城的互动，在开放中提升协同发展水平。

(三)坚持绿色发展，建设生态文明新家园。

坚持“依法施治，精准施策，全民参与”，以最大的决心、最实的措施，坚决打好生态环保治理攻坚战。把大气污染治理放在突出位置，认真落实防治雾霾十大措施，系统加强燃煤控制、工业排放、城市扬尘、机动车尾气、挥发性有机物综合治理，做到源头严防、过程严管、事故严惩，坚决遏制重污染天气频发态势。实施工业绿动力计划，大力发展节能环保产业，加快淘汰落后产能，严格落实重点行业脱硫、脱硝、除尘改造任务。积极优化能源结构，加快太阳能等清洁能源使用，到 2017年全市将压减燃煤93万吨。抓好水污染防治，严格治理企业非法排污行为，加强水生态保护和水资源管理。强化农业面源污染防治，发展生态循环农业，促进土壤质量明显提升。实施大规模国土绿化行动，新建一批湿地公园，创建国家森林城市。

(四)坚持协调发展，增强发展均衡性。积极构建城乡协同发展新格局，坚持做大做强中心城区，严格控制城市规划，提高城市管理水平，有序引导中心城区向东拓展，加快德陵一体化进程，增强中心城区对周边县城的辐射带动作用。抓住国家政策机遇，用五年时间，基本完成中心城区、各县城棚户区和城中村改造任务。积极推进新型城镇化，有序推进外来务工人员市民化、城中村和城边村原有居民市民化、农村就地转移就业人口市民化，全面推行居住证制度，逐步实现教育、医疗、社保、就业服务和住房保障等城镇基本公共服务常住人口全覆盖。继续推进实施“两区同建”，合理把握节奏，统筹资源要素，更加注重可持续性，更加注重配套质量和群众满意度，大力突破农业产业园区建设，不断探索新的融资模式，持续推进农村新型社区建设。实施“乡村记忆”工程，加强乡村文化保护，加大对历史文化建筑、特色街区村落、非物质文化遗产的保护。加快美丽乡村建设，持续开展村容村貌整治，2018年底完成59.7万农户无害化卫生厕所改造。

(五)坚持共享发展，让德州人民生活更美好。坚持以人民为中心，使德州广大群众在共建共享中有更多获得感。全面实施精准扶贫、精准脱贫是党中央作出的重大部署和庄严承诺，是全面建成小康社会的重要内涵，我们要以高度的政治责任感，全力以赴做好精准扶贫工作，动员一切社会资源和力量，帮助16万困难群众走出贫困，稳定实现贫困人口“两不愁、三保障”，力争“两年脱贫、一年兜底、两年巩固”，提前完成脱贫任务。

加大民生保障力度，关注群众切身利益，加快解决上学难、看病难、停车难、老旧小区生活难等问题，集中力量实施突破。教育方面，五年内新建、扩建中小学 265所，改造薄弱学校891所，新增学位20.3万个，力争两年内基本解决“大班额”问题，三年内完成全市所有薄弱学校改造任务。大力加强师资队伍建设，全面改善农村教学点教学力量不足的状况。大力发展职业教育。卫生方面，继续提升中心城区医疗水平，五年内市级公立医院床位由2463张增加到6000 张以上。支持各县(市、区)公立医院对外开展合作，大力培养中医药人才，加强各级中医院建设。老旧小区整治方面，中心城区三年内完成所有“三无小区”改造，探索新的物业管理模式，各县城全面开展老旧住宅小区和背街小巷整治改造。增加城市停车场数量，鼓励社会资本进入，合理规划，优先布局充电桩等设施。

完善社会治理体系，健全群众利益诉求表达、社会矛盾排查预警和调处化解机制，深入开展平安德州建设。加强食品药品监管，强化安全发展理念，全力抓好安全生产工作，确保人民安居乐业、社会安定有序。高度关注弱势群体，完善城乡低保、医疗、教育、住房、就业等社会救助体系。大力发展文化事业，完善公共文化服务体系，实现基层综合性文化服务中心全覆盖。全面落实民族宗教政策，深入开展民族团结进步教育，保障妇女、未成年人和老年人合法权益，关心关爱农村留守儿童，提升市民科学素质，加快残疾人事业发展。

(六)坚持深化改革，增强协同发展新动力。

坚定不移地推进重点领域改革，靠改革增活力、解难题、促发展。抓好行政体制改革，进一步转变政府职能，推进简政放权、放管结合、优化服务，创造全省一流的政务环境;加快事业单位分类改革，逐步实现学校、医院和科研院所去行政化;深化投融资体制改革，完善多层次金融市场体系，防止发生区域性系统性风险。加快国有企业改革，建立现代企业制度，优化国有资本布局，推动国有资产监管由管资产向管资本转变，实现市级经营性国有资产监管全覆盖。推进农村综合改革，深化农村土地征收、集体产权制度等改革。加快司法体制改革和社会领域改革，深入推进综合行政执法、基础教育、医药卫生和户籍制度等项改革。

各位代表，“十三五”规划的蓝图已经绘就，我们满怀信心，激情澎湃!相信在中共德州市委的坚强领导下，在全市人民的共同努力下，我们一定能够顺利完成既定目标，推动德州在协同发展的道路上再创辉煌!三、2016年重点工作安排

今年是“十三五”的开局之年，我们将认真落实中央供给侧结构性改革的总体要求，做好去产能、去库存、去杠杆、降成本、补短板等项工作。主要预期目标是：地区生产总值增长8%左右，固定资产投资增长13%以上，公共财政预算收入增长7%以上，社会消费品零售总额增长11%以上，进出口保持稳定。城镇登记失业率控制在3.5%以内。城镇和农村居民人均可支配收入分别增长9%和10%左右。常住人口城镇化率、户籍人口城镇化率分别达到53.48%、 44%。全面完成省下达的节能减排约束性指标。具体工作中，重点抓好以下六个方面：

(一)加快建设协同发展示范区。统筹实施京津冀协同发展和“一区一圈一带”战略，紧盯京津冀协同发展“十三五”规划，抓好省委、省政府政策措施落地。全年签约落地产业项目300个左右，引进亿元以上项目到位资金600亿元以上。引导各县(市、区)立足特色化、差异化，重点培植发展1至2个产业集群，增强产业集中度。突出抓好北汽、奇瑞新能源汽车等龙头产业项目，年内实现新车下线。加快实施北京联东U谷产业园、天津食品集团产业基地、河北华日集团高端家具产业园等重点产业项目。积极引入创新资源，建设中关村海淀园齐河科技城、山东石墨烯产业研究院、山东大学新材料与智能装备研究院。深化教育、医疗、文化等领域的合作，加强与首都医科大学、中国出版集团、北京交通大学、天津美术学院、中国供销集团、首农集团、荣宝斋的合作。加强专业招商能力，组建智能装备制造、生物技术等五个战略性新兴产业办公室，实现由招商引资向产业组织转型、政策优惠向环境优化转型、政府主导向政府引导转型。组织开展好京津冀鲁资本交易大会、技术成果交易大会、“智汇德州”人才创新创业周三项重点活动，深化与周边城市的合作。

(二)着力抓好经济稳增长。突出抓大扶强，落实“3个100”企业培植计划，设立1亿元的工业发展扶持资金，加大对重点产业的扶持力度，组织实施通裕重工、玲珑轮胎等150个工业转型升级项目，技改投入达到工业投入的60%以上，高新技术产业产值占比达到30%。抓好威讯半导体、华鲁恒升、大国重器等 102个市级重点项目，确保早开工、早投产，完成年度投资411亿元。推进大众创业、万众创新，新增市场主体5万个，建设企业科技创新平台120个，转化科技成果200项以上。

牢固树立“政府创造环境，企业创造财富”的理念，大力优化企业发展环境。进一步减少企业制度性交易成本，减少审批环节，清理“红顶中介”，引入竞争机制，降低企业成本。全面优化金融生态环境，增强金融机构信心，发挥好政策性平台作用，及时帮助企业化解因担保链、担保圈而形成的风险。发挥好已组建的十大产业联盟的作用，加强产业整合，加大对企业家队伍的引导和服务，打造产业集群。积极化解过剩产能，妥善处置僵尸企业，对资不抵债、扭亏无望的企业，积极推进重组整合或退出市场。发挥好安全生产和环保标准的倒逼作用，对安全生产存在重大隐患、环保不能达标排放的企业，一律取消政策性支持。促进房地产业健康稳定发展，采取多种措施，积极帮助企业减少库存。一刻不放松地抓好安全生产工作，落实好“专家查隐患，企业抓整改，政府抓督导”要求，开展好安全生产“保底行动”，确保生产安全、社会安全。

(三)加快“放心农场”建设，推进农业现代化。以供给侧结构性改革为导向，加快推进我市农业发展方式转型，建设一批市级“放心农场”，千方百计促进农民增收致富。牢固树立产业思维和效益意识，加快现代农业体系建设，高水平编制完成《国家现代农业示范区建设总体规划》，出台支撑现代农业发展的政策体系;继续促进土地合理流转，实现适度规模化经营，提高农业专业合作社的能力;推动区域性农产品交易中心建设，建成一批大型农产品批发市场、物流配送中心，拓展渠道，使德州的优质农产品能顺利进入高端消费市场;大力推广设施农业建设，引导农民调整种植结构，在确保粮食安全的前提下，加大经济作物种植。支持临邑县与央企合作，率先建设工业级“超级农业大棚”，发挥好引领作用。支持平原县、齐河县分别与世界500强德国巴斯夫公司、美国雅培公司合作建设生态农用地膜和高端乳制品产业基地，支持各县(市、区)积极推进现代农业国际合作，大力引进先进技术和跨国企业。加强农产品质量安全监管体系，农产品质量安全抽检合格率稳定在96%以上;强化农业品牌意识，“三品一标”认证面积占食用农产品总面积的比重稳定在60%以上。支持地方涉农产业龙头企业做大做强，加快乐陵农高区建设，积极争取国家农业大数据中心落户德州，启动建设区域性农业技术交易中心。

(四)全面做好民生和社会事业。坚定不移把精准脱贫作为第一民生工程，搞好精准识别，切实摸清家底、选好脱贫路径，坚持一村一策、因户制宜，把扶贫资金、项目落实到户到人。把产业扶贫作为重要的脱贫方式，引导贫困群众发展特色高效种养业，加强技能培训，搭建就业对接平台，支持有劳动能力的贫困人口就业创业。扎实推进农村低保线与扶贫线“两线合一”，各县(市、区)低保线不低于国家扶贫标准线。进一步完善居民大病保险制度，贫困人口最高支付限额提高到每年50万元。强化教育扶贫，推进从学前教育到研究生教育阶段家庭经济困难学生资助的全覆盖，阻断贫困的代际传递。对755个省扶贫工作重点村，大力加强道路、水利、电力等基础设施建设，改善生产生活条件。今年市财政安排1亿元专项资金用于扶贫，力争完成70%重点贫困村、贫困人口的脱贫任务。

认真落实中央、省各项惠民政策，继续办好民生实事。大力改善办学条件，全年新建改建学校105处，增加学位8.7万个。德州高级师范学校启动建设。开工建设市残疾人康复中心、市中医院病房楼、市妇女儿童医院病房楼，启动市东部医疗中心建设，完成市人民医院新门诊楼主体工程，全面提高公共卫生服务水平。认真落实创业扶持政策，新增城镇就业5.5万人。实施全民参保登记计划，推动机关事业单位养老保险制度改革，居民医保住院报销比例提高2个百分点。大力发展养老事业，新增养老床位4000张。投资37.95亿元对中心城区棚户区实施改造，建成保障性住房8000套。投资3.5亿元对73个老旧小区和背街小巷实施改造，完善道路、排水、照明、绿化、供热等条件。落实困难残疾人生活补贴和重度残疾人护理补贴，年内为70名听障儿童实施人工耳蜗植入手术。全面落实两孩政策。加强公共文化体育设施建设，乡镇综合文化站全部达到国家三级以上标准，村级综合性文化服务中心和健身路径分别达到60%和80%。完善立体化治安防控体系，确保社会安全稳定。

进一步提高新型城镇化水平。中心城区投资152亿元，重点实施广川大道北延、南运河污水管网迁建等62个城建项目，完善城市供排水、供热、燃气、垃圾处理等功能设施。推进公交优先发展，新增新能源公交车350辆。降低外来人员落户门槛，提高棚户区改造货币化安置比例。巩固国家卫生城市创建成果，完善城市管理和服务，提高精细化管理水平。扎实推进智慧城市建设，完善智能交通、数字城管等公共平台。规划建设海绵城市，提高防洪排涝减灾能力。开展中心城区违章建设大整顿行动，严厉打击违法建设行为。实施县城和示范镇提升行动，改善农村人居环境，建设无害化卫生厕所14万户，改造农村危房3223户，启动农村供暖改造工程。加快石济客专等重点交通工程建设，完成农村公路生命安全防护工程建设任务。

(五)统筹抓好各重点领域改革事项。按照“改革政策要实”的要求，加快重点领域改革，着力破解制约德州发展的关键问题。持续深化投融资体制改革，整合城市有效资源，搭建新的融资平台，建立市场化运行机制，形成新的融资模式。加快组建国有资本运营公司，市财金投资公司、融资性担保公司挂牌运营，城市建设、交通、土地储备、医疗、教育等平台公司加快筹备，国有融资担保机构实现县域全覆盖，加快推进48个PPP项目，承接好政策性银行信贷投放。加快推进农信社银行化改革，年内11家农信社全部完成改制任务。深化财税体制改革，优化财政支出方式，控制政府债务规模，积极采用融资租赁等方式加强与金融机构的合作。所有使用财政资金的部门，要全部公开预决算。深化国税地税征管体制改革，完善联合办税机制，落实“营改增”扩围等结构性减税政策。积极探索我市完善和拓展土地增减挂钩试点，有序推进禹城市征地制度改革试点，完成好国家试点任务。启动城市公立医院综合改革，深化基础教育综合改革，完成党政机关公车改革任务。

(六)强力推进以大气污染防治为重点的生态环保工作。集中力量打好大气污染防治攻坚战，全面做好十项重点工作，力促空气质量明显好转。上半年出台我市第一部政府规章《德州市大气污染防治管理规定》，健全依法治理体系。修订《德州市重污染天气应急预案》，强化预案演练，提高城市应急处置能力。完善环境监测体系，建立数字预警模式，健全联席会商机制，提高对雾霾的预判能力。严格投资项目节能环保准入，严控高耗能、高排放行业新增产能，编制建设项目准入负面清单，推动重化企业向专业园区聚集。抓住控煤这个主要矛盾，全力抓好大型燃煤机组和锅炉超低排放改造，实施小锅炉达标工程，中心城区淘汰、拆除10吨以下锅炉，各县(市)逐步淘汰10吨以下工业燃煤锅炉。坚决管住散煤，全面推广清洁煤使用，建立清洁煤配送体系，进一步扩大中心城区和县城集中供热面积。全面依法打击、取缔非法加油站点和各类土小企业，加快提升燃油品质。全面落实扬尘治理“六化”要求，持续加大城市机动车尾气、餐饮油烟、挥发性有机物的治理力度。统筹安排好全市工业企业错峰生产和调休，减少冬季排放压力。提升监测监控能力，建筑拆迁工地、商混企业、大型堆场、20吨以上锅炉全部安装在线监控设施。深化流域污染防治，实施工业污染源全面达标排放计划，确保主要河流水质稳定达到地表水Ⅴ类标准。加大生态修复力度，全年新增造林20万亩、植树 2500万株，集中实施一批景观绿化、生态湿地提升工程。加强环境信息公开，加大对环境违法单位曝光力度，对环境违法犯罪行为严查重处、绝不姑息。四、汇聚协同发展的强大力量

各位代表，做好“十三五”工作，全面建成小康社会，是我们对全市人民的庄重承诺，我们将秉承为民、务实、清廉要求，全面建设法治政府、效能政府和廉洁政府。

深入推进依法行政。全面贯彻落实《法治政府建设实施纲要》，坚持法无授权不可为、法定职责必须为。认真执行市人大及其常委会的各项决定，定期报告市政府工作。积极接受市政协民主监督，听取政协委员的工作建议。主动接受社会监督、舆论监督，健全重大行政决策公众参与、专家论证、风险评估、合法性审查制度机制，严格按照法定权限和程序履行政府职能。

全面提高政府服务效能。大力倡导从严从实、奋发有为的精神，本着对群众高度负责的态度，着力提高各级干部的执行能力和创新能力，坚决破解不会为、不愿为、不敢为问题，坚决克服庸懒散现象，坚决防止损害群众利益的行为。认真落实政府服务承诺，做到言必信、行必果。真心实意为群众办实事、做好事、解难事，全心全意为人民服务。

切实加强廉洁政府建设。坚决执行党风廉政建设和反腐败的各项要求，坚决落实党风廉政建设主体责任，加强廉政教育和制度建设，强化公共资源交易监督管理，盯住重点领域和关键环节，对腐败问题绝不手软。坚决把纪律和规矩挺在前面，严格落实中央“八项规定”精神，持之以恒纠正“四风”，以实际行动赢得人民群众的信任与支持。

各位代表，实施协同发展战略，建设协同发展示范区是全市人民共同的事业。做好今年的各项工作，必须巩固和发展最广泛的爱国统一战线，认真听取各民主党派、工商联和无党派人士的意见建议;全面落实党的知识分子、民族、宗教、侨务等政策，加强与港澳台同胞和海外侨胞的交流合作。必须充分发挥工会、共青团、妇联等群团组织联系群众、服务群众、动员群众的作用;扎实开展双拥共建，巩固和发展军政军民团结，推动军民深度融合发展。必须全面落实尊重劳动、尊重知识、尊重人才、尊重创造方针，激发人民群众建设协同发展示范区的主人翁意识。

各位代表!时代赋予重任，人民寄予厚望，我们深感责任重大、使命光荣。让我们紧密团结在以习近平同志为总书记的党中央周围，在中共德州市委的坚强领导下，解放思想，抢抓机遇，努力建设协同发展示范区，为顺利完成“十三五”规划目标、全面建成小康社会而努力奋斗!
